# Intraluminal eradication via transmural supply blocking, a novel concept for the treatment of esophageal and gastric varices by endoscopic ultrasound-guided perforating vein blocking

**DOI:** 10.1093/gastro/goaf069

**Published:** 2025-08-06

**Authors:** Di Zhang, Lei Lei, Chao Zhou, Xiaogang Liu, Chao Huang, Hongxue Lu, Guanyu Zhou, Pu Wang

**Affiliations:** Department of Gastroenterology and Hepatology, Sichuan Provincial People’s Hospital, School of Medicine,University of Electronic Science and Technology of China, Chengdu, Sichuan, P. R. China; Department of Gastroenterology and Hepatology, Sichuan Provincial People’s Hospital, School of Medicine,University of Electronic Science and Technology of China, Chengdu, Sichuan, P. R. China; Department of Gastroenterology and Hepatology, Sichuan Provincial People’s Hospital, School of Medicine,University of Electronic Science and Technology of China, Chengdu, Sichuan, P. R. China; Department of Gastroenterology and Hepatology, Sichuan Provincial People’s Hospital, School of Medicine,University of Electronic Science and Technology of China, Chengdu, Sichuan, P. R. China; Department of Gastroenterology and Hepatology, Sichuan Provincial People’s Hospital, School of Medicine,University of Electronic Science and Technology of China, Chengdu, Sichuan, P. R. China; Department of Gastroenterology and Hepatology, Sichuan Provincial People’s Hospital, School of Medicine,University of Electronic Science and Technology of China, Chengdu, Sichuan, P. R. China; Department of Gastroenterology and Hepatology, Sichuan Provincial People’s Hospital, School of Medicine,University of Electronic Science and Technology of China, Chengdu, Sichuan, P. R. China; Department of Gastroenterology and Hepatology, Sichuan Provincial People’s Hospital, School of Medicine,University of Electronic Science and Technology of China, Chengdu, Sichuan, P. R. China

## Introduction

Endoscopic ultrasonography (EUS)-guided therapy demonstrates enhanced precision and therapeutic efficacy in managing esophageal and gastric varices compared to conventional white-light endoscopic approaches. However, both approaches are based on the same underlying therapeutic principle: achieving variceal obliteration instead of direct intervention on the varice’s primary blood supply source. If precise blockade targeting the blood supply to varices could be implemented, particularly targeting the transmural perforating veins that are most amenable for EUS-guided access, more profound variceal eradication could be achieved with potential reduction in recurrence rates. Given the high-resolution mapping of portal venous system vasculature that is achievable via EUS, perforating veins responsible for the blood supply of varices could be precisely identified and located (Supplementary Materials 1). Subsequent EUS-guided embolization using coils or glue can be utilized to realize this ideal therapeutic concept. Here, we introduce a novel therapeutic strategy for upper gastrointestinal varices termed Intraluminal Eradication via Transmural Supply Blocking (IETSB), which operates by blocking transmural perforating veins and thus preventing portal pressure from entering the digestive tract to form varices. Two representative cases demonstrating the preliminary feasibility of this methodology are presented below.

## Case report

### Case 1

**Figure 1.**
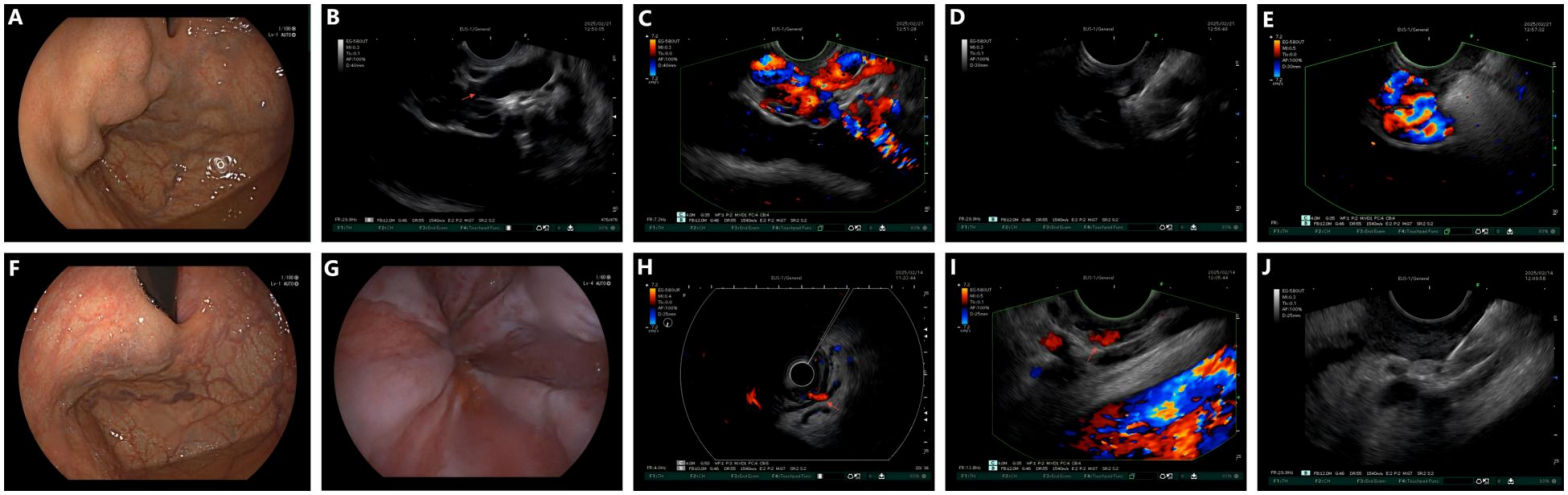
Illustration of case 1 and case 2. (A) Gastric varices under white light view. (B) Gastric varices under EUS view (arrow: perforating vein demonstrated by muscularis propria discontinuity). (C) Gastric varices under EUS Doppler scan. (D) EUS-guided coil and glue injection at the orifice of perforating vein. (E) No blood signal within perforating vein under EUS Doppler scan. (F) 20 minutes after, the gastric varices significantly reduced in size (G) Esophageal varices under white light view. (H) Radial EUS scan (arrow: perforating vein with red Doppler signal, indicating blood inflow). (I) Linear EUS scan (arrow: perforating vein with red Doppler signal, indicating blood inflow). (J) EUS-guided glue injection at the orifice of perforating vein.

A 74-year-old female presented with mild abdominal discomfort for 20 days, exhibiting no onset of upper gastrointestinal bleeding or other major complications. Computed tomography demonstrated liver cirrhosis with portal hypertension. Upper gastrointestinal endoscopy revealed isolated gastric varices (Figure 1A). Laboratory tests showed hemoglobin of 108 g/L without coagulopathy or hepatic dysfunction.

Transesophageal-approached EUS scan identified a 5.2 mm width transmural perforating vein at the gastric fundus, connecting the gastric varices to extra-luminal veins through muscularis propria discontinuity (Figure 1B and C). Vascular tracing confirmed the perforating vein originated from the short gastric vein that served as the primary feeder.

Intra-variceal blood flow velocity was assessed as 13 cm/s before EUS-guided treatment. EUS-guided embolization was then performed by using a 22G needle to deploy two 6-mm coils with 1 mL glue at the orifice of perforating vein (Figure 1D and E). Immediate variceal shrinkage was observed within 20 minutes (Figure 1F). Post-procedural Doppler scan confirmed complete flow cessation in the perforating vein, with intra-variceal blood flow velocity decreasing to 4 cm/s.

At 1-month follow-up, complete variceal resolution was observed, the surface mucosa was intact without any ulceration (see Supplementary Materials 2). No adverse events occurred.

### Case 2

A 39-year-old male with alcoholic cirrhosis was admitted with first-time bleeding due to esophageal varices and gastric varices on the lesser curvature (gastroesophageal varices type 1 according to Sarin classification) (Figure 1G). Laboratory tests revealed hemoglobin 121 g/L without hematologic, hepatic, or coagulation abnormalities.

First, by using radial EUS, a 2.8 mm width transmural perforating vein at the cardia level was identified, displaying a characteristic red Doppler flow signal that indicates a extramural-to-intraluminal inflow direction (Figure 1H). Then by using linear EUS, we traced the left gastric vein all the way to the perforating vein (Figure 1I). Intra-variceal blood flow velocity was assessed as 5 cm/s before treatment.

EUS-guided embolization was performed via 22G needle to inject 0.5 mL glue at the orifice of the perforating vein (Figure 1J), utilizing a pre-/post-flush technique (1–2 mL liquid) to prevent needle tract occlusion. Immediate doppler scan confirmed complete embolization of the perforating vein and 0 cm/s intra-variceal blood flow velocity. Partial variceal shrinkage was observed within 20 minutes.

At 1-month follow-up, endoscopic evaluation demonstrated significant esophageal varices resolution without procedure-related mucosal injury (see Supplementary Materials 2). No complications occurred.

EUS was performed using SU9000 system (Fujifilm CO., LTD) with EG-580 UT/UR scopes. Embolization materials included: 22G needle and Coil (Cook Medical CO., LTD), Cyanoacrylate glue (B Braun Surgical, S. A.).

## Discussion

Due to liver cirrhosis, blood flow in the portal system is obstructed. Increased portal pressure drives a portion of blood through the natural portosystemic shunt into the systemic venous system. Meanwhile, as portosystemic shunt is insufficient to release all portal hypertension, another portion of blood is also forced by the portal hypertension to reflux into the veins that originally serve as drainage vessels of the digestive tract [1–3], and these blood that flows back toward the digestive tract ultimately enters the lumen through perforating vein to facilitate the formation of digestive varices. This pathophysiological mechanism reveals why the current endoscopic therapies, which only focus on obliterating varices seen in the digestive tract without blocking the underlying source of blood supply, yield suboptimal efficacy.

EUS enables precise localization of varices and surrounding vasculature and thus holds groundbreaking promise for redefining current standards in variceal treatment. However, current EUS-guided therapeutic approaches underutilize its technological superiority, and still focus solely on improving injection accuracy of hemostatic agents and ensuring appropriate dosage as compared to white-light endoscopic therapy, instead of addressing the root cause-occluding the blood supply of varices. Consequently, despite achieving some therapeutic efficacy enhancement and ectopic embolism risk reduction, this kind of preliminary and superficial EUS applications still retains the post-procedural variceal rebleeding rates at 14%–30% [4].

However, the advantages of EUS could be maximized by adopting IETSB strategy, which changes the treatment concept from passive variceal obliteration to active hemodynamic intervention. When following IETSB strategy, targeted occlusion of transmural perforating veins under EUS guidance deprives intraluminal varices from the inflow of its blood supply [5], thus promoting variceal regression, decreasing recurrence risk and reducing the amount of hemostatic agent requirements. Moreover, the spontaneous portosystemic shunts are naturally preserved, and broader hemodynamics are not affected by implementing IETSB strategy. Therefore, this treatment does not cause a further increase in portal pressure as seen in balloon-occluded retrograde transvenous obliteration, nor does it lead to hepatic encephalopathy as seen in transjugular intrahepatic portosystemic shunt. The portal pressure is eventually prevented from entering the gastrointestinal lumen and stays outside, and thus, the intraluminal eradication of varices is achieved. The significance of this lies in the inherently lower hemorrhage risk of high-pressured extraluminal veins, which is due to absent exposure to irritants like gastric acid, food, as well as the mechanical stress during gastrointestinal peristalsis, and so on.

Regarding the technique, first, we need to understand where the blood supply for varices comes from. Gastric fundal varices typically derive from the short and/or posterior gastric veins, with transmural perforating veins commonly localized to the fundus, whereas the esophageal varices and lesser curvature gastric varices predominantly originated from the left gastric and paraesophageal veins [3]. Second, the treatment strategies for varices in different locations are also different. Given the rapid flow velocities typically observed within isolated gastric varices, empirical observations suggest that slowly releasing coil facilitates the formation of stable embolic aggregates, thereby ensuring reliable occlusion of perforating vein. Optimal outcomes can be achieved by selecting coils with diameters slightly exceeding the width of the orifice of the target perforating vein, supplemented by adjunctive glue may enhance blocking efficacy. Meanwhile, esophageal varices and gastroesophageal varices type 1 demonstrate slower flow dynamics and a narrower width of perforating vein. For these subtypes, either small-diameter coils or glue monotherapy could be an appropriate approach.

In addition, before IETSB therapy, it is crucial to distinguish whether a perforating vein is a blood supply vessel or a drainage vessel by judging the Doppler signal color or by contrast injection. If a perforating vein is identified as a draining vein, then this is not for IETSB intervention, and conventional complete variceal obliteration therapy should be done. Coils can still be deployed to occlude the transmural perforating vein orifice, as this vein may drain into a portal-systemic shunt, blocking it first could reduce the risk of ectopic embolism caused by subsequent injection of glue. This approach can represent an improvement over current EUS-guided methods that do not consider precise coil placement locations.

In conclusion, the IETSB strategy may hold transformative potential for gastrointestinal varices management by fundamentally addressing the limitations of current therapies. This study, while providing preliminary proof-of-concept validation, remains limited by sample size and the absence of long-term data. Future research is warranted both on treatment technique advancement that can achieve consistently stable occlusion of perforating veins, as well as on long-term effectiveness and safety demonstration.

## Supplementary Material

goaf069_Supplementary_Data
