# Supplementary material for: Intraluminal eradication via transmural supply blocking, a novel concept for the treatment of esophageal and gastric varices by endoscopic ultrasound-guided perforating vein blocking
Source: Gastroenterol Rep (Oxf). 2025 Aug 6;13:goaf069. doi: 10.1093/gastro/goaf069 (PMC12342188; doi:10.1093/gastro/goaf069)
Supplement: goaf069_Supplementary_Data [file goaf069_supplementary_data.zip › supplementary_materials_1-_illustration_of_EUS_scan_of_perforating_vein.docx]

**Supplementary materials 1- illustration of EUS scan of perorating vein**

As shown in Figure S1.1, At the level of the cardia, EUS clearly demonstrated the His angle, including the muscularis propria of the esophageal wall and gastric fundus, GV within the gastric lumen, dilated veins outside the gastric wall and discontinuous appearance in the muscularis propria of the gastric fundus which is a typical feature of transmural perforating veins. Also at the level of the cardia, it is not difficult to detect the presence of perforating veins in the EV by using radial EUS (Figure S1.2). With linear array EUS, we can trace the left gastric vein from its confluence with the splenic vein all the way up to the esophageal vein. By carefully scanning the dilated esophageal vein as it gets closer to the esophageal wall, perforating vein that connect the EV and extraluminal paraesophageal vein can be identified as shown in Figure S1.3.

Additionally, we can assess the direction of blood flow within the perforating veins that are not parallel to the probe by observing the color of Doppler signals (As long as the perforating blood vessels are relatively perpendicular to the wall of the digestive tract, Doppler scanning shows a basic pure red color indicating blood inflow, and a basic pure blue color indicating blood outflow) or by injecting SonoVue contrast agent, thereby determining whether the perforating veins are feeding vessels or draining vessels.


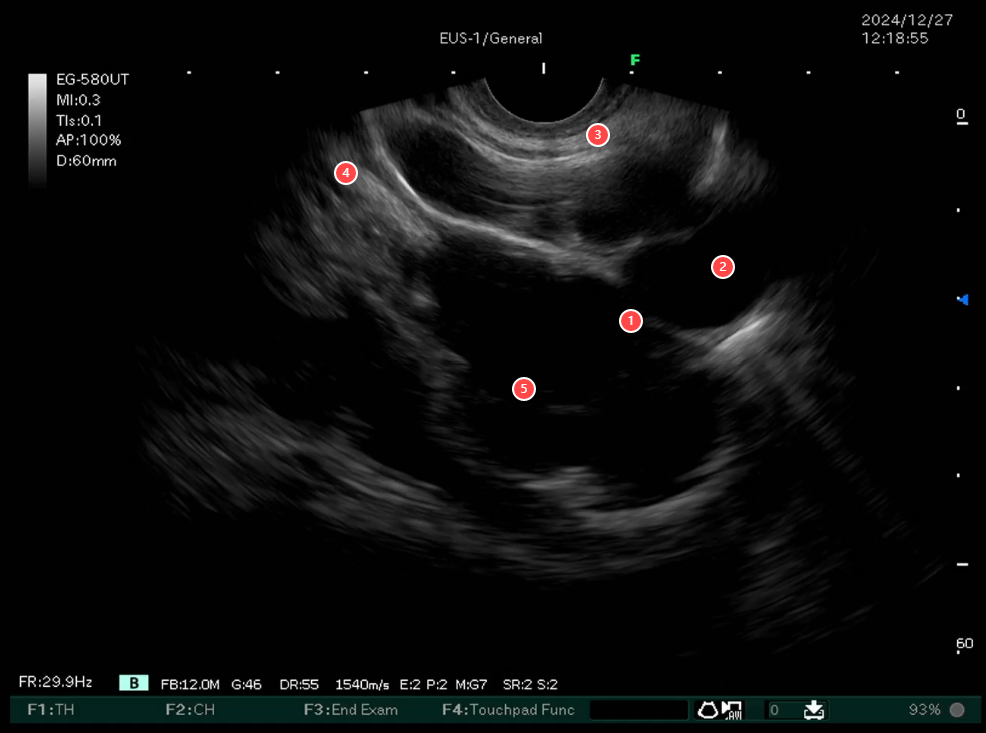


Figure S1.1

EUS scan for gastric varices 1. perforating veins (muscularis propria discontinuity), 2. Extraluminal veins, 3. lamina propria of esophagus, 4. His angle and lamina propria of Gastric fundus 5,Gastric varices


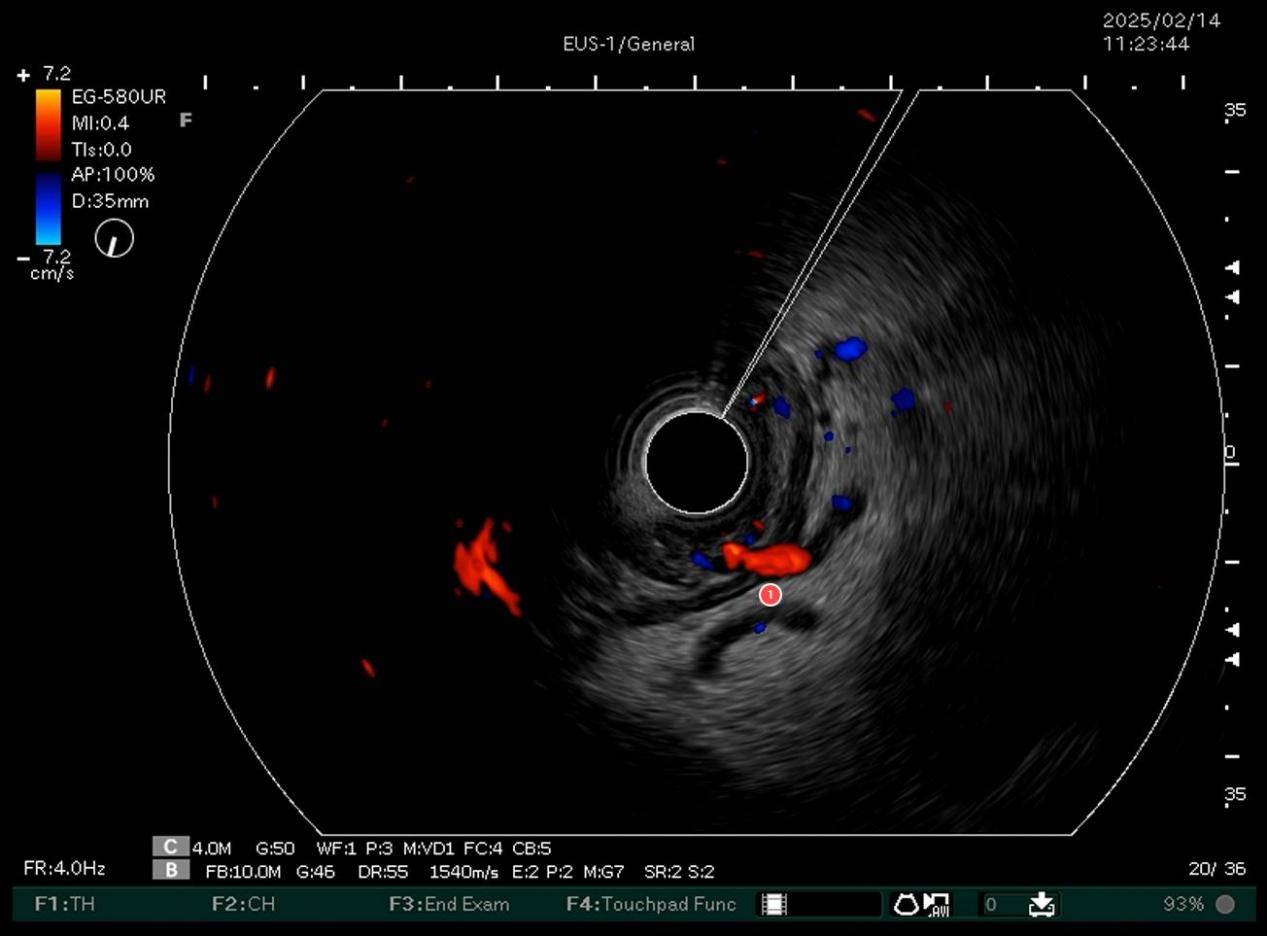


Figure S1.2

Radial EUS sacan for perforating vein

1. perforating veins


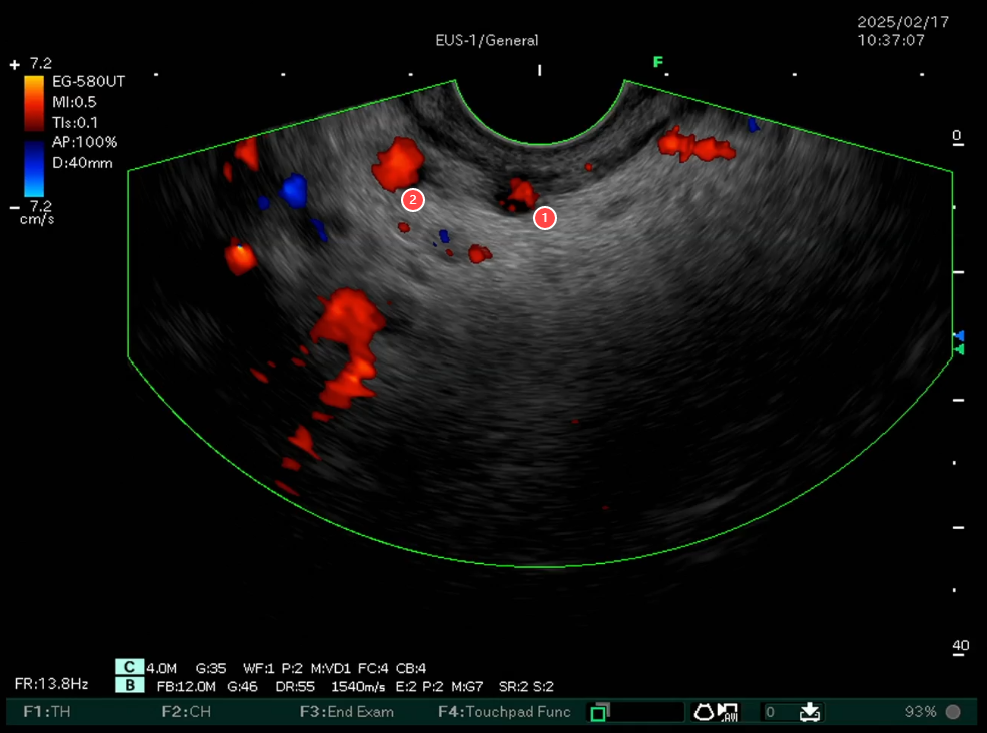


Figure S1.3

EUS Doppler scan for esophageal varices 1. perforating vein, 2. paraesophageal vein
