# Supplementary material for: Intraluminal eradication via transmural supply blocking, a novel concept for the treatment of esophageal and gastric varices by endoscopic ultrasound-guided perforating vein blocking
Source: Gastroenterol Rep (Oxf). 2025 Aug 6;13:goaf069. doi: 10.1093/gastro/goaf069 (PMC12342188; doi:10.1093/gastro/goaf069)

**Supplementary materials 2- 1 month-follow-up of 2 cases**

Figure S2.1-S2.3 show the results of up GI endoscopy and EUS of patient 1 after 1 month. It can be seen that GV disappeared completely, the surface mucosa was smooth and complete without any ulcer that can be usually seen after glue injection therapy. The small bulge was the complex of coil and glue. By EUS we can see that the slightly hyperechoic signals of the coil and glue are stable at the original perforating vessel, part of which protrudes into the lumen to form the bulge seen by white light endoscopy (Similar to a cork blocking a hole), and part of which protrudes into the blood supply vein outside the lumen. There is no blood flow signal in the lumen and the blood flow signal of the extramural blood supply vein was also alleviated.

Figure S2.1 white light image


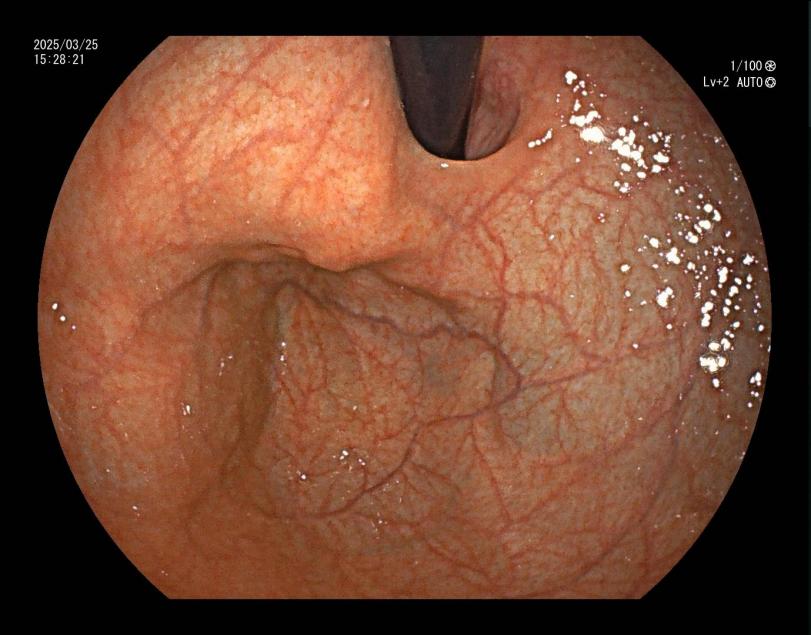


Figure S2.2 EUS scan


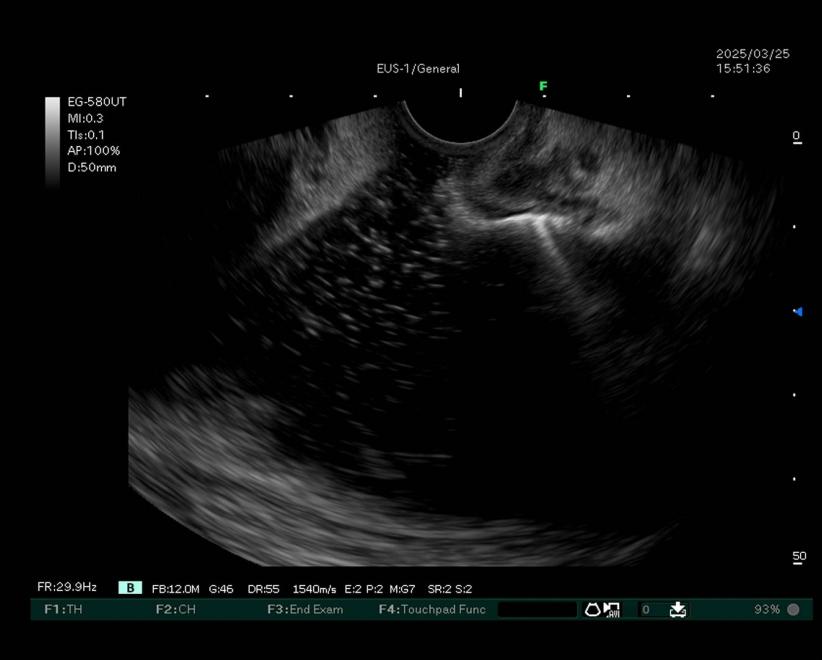


Figure S2.3 Doppler scan


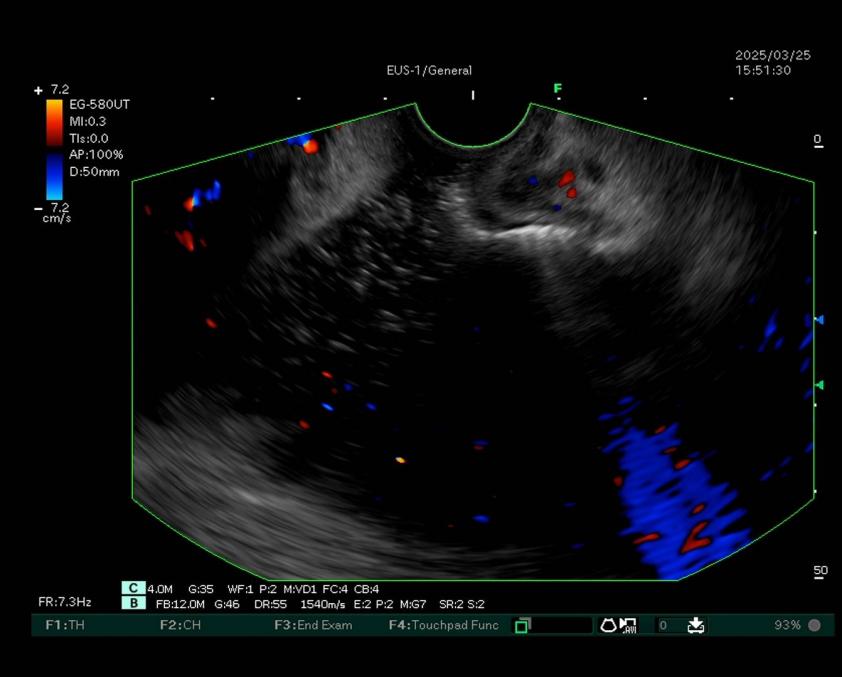


Figure S2.4-2.5 show the results of up GI endoscopy and EUS of patient 2 after 1 month.

Figure S2.4 white light image

presents the upper gastrointestinal endoscopic findings of Patient 2 at the 1-month follow-up, demonstrating significant regression of esophageal varices.


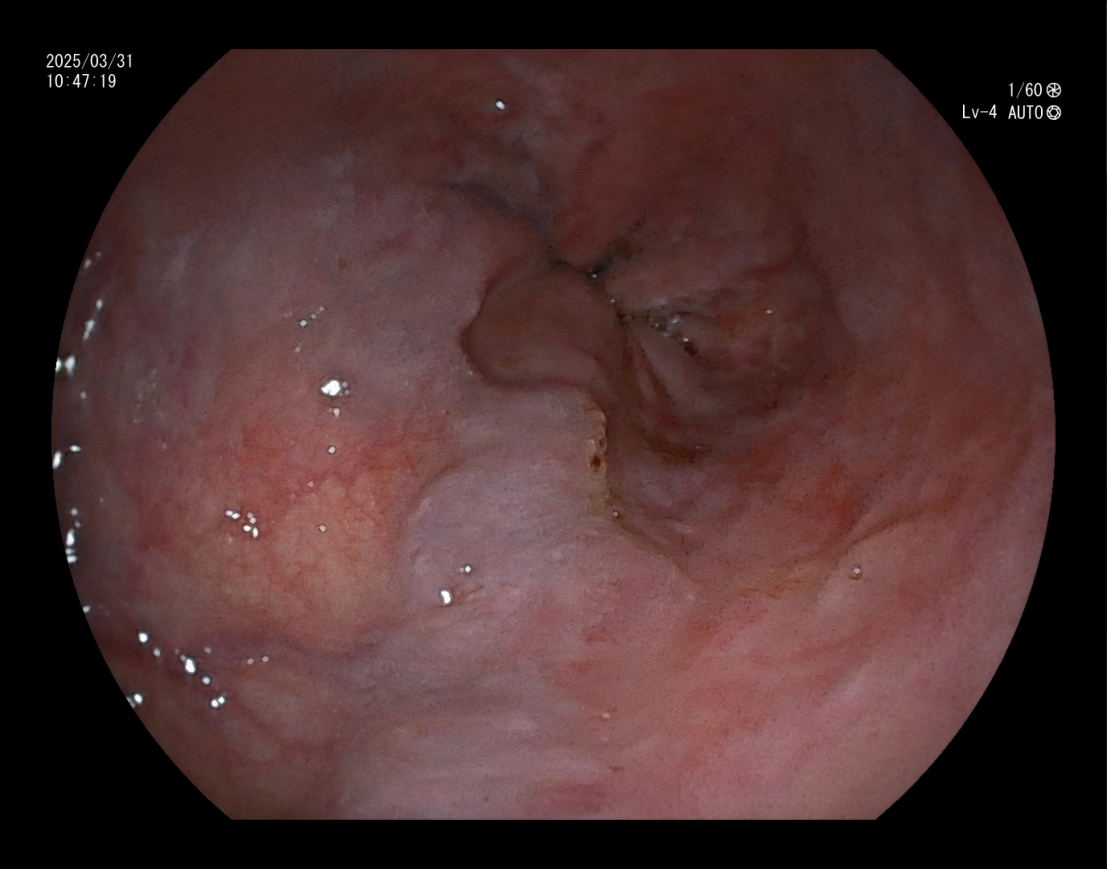


Figure S2.5 Radial EUS imaging

Radial EUS imaging revealed persistent retention of glue at the vascular perforation site. Doppler evaluation further confirmed complete obliteration of extraluminal blood flow, indicating successful hemodynamic isolation achieved by the precise glue injection


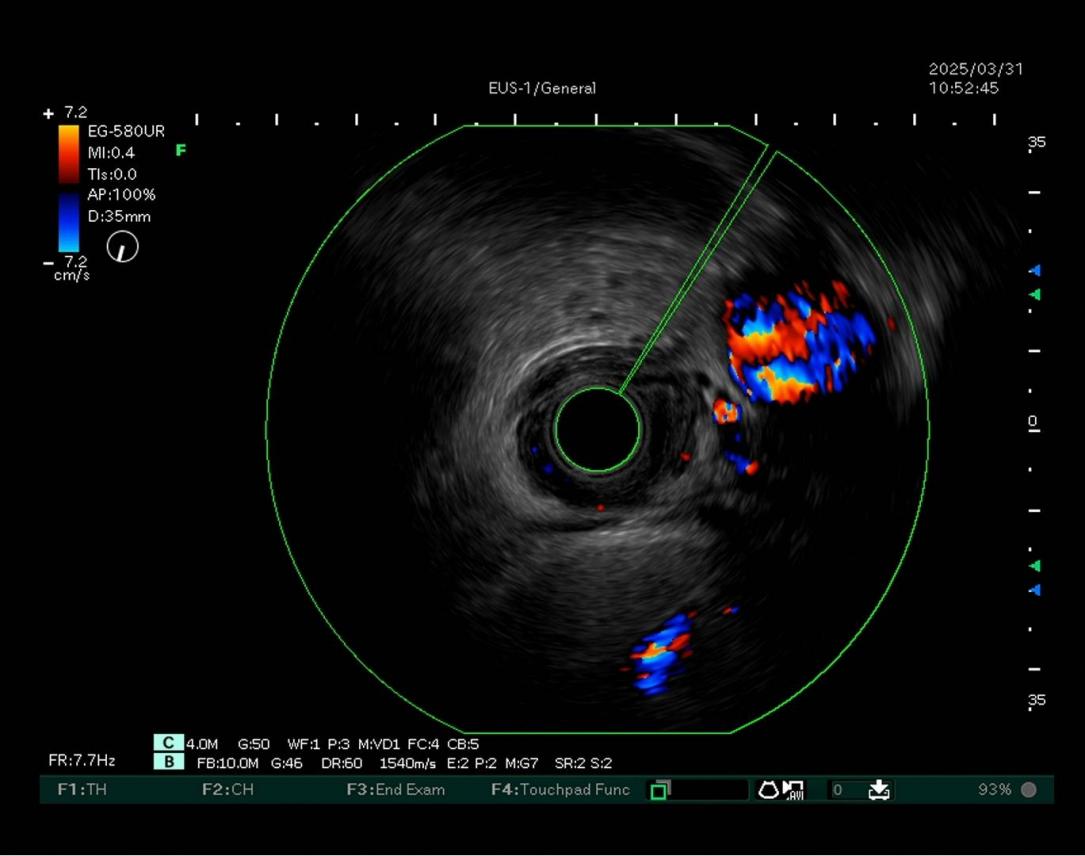

Supplement: goaf069_Supplementary_Data [file goaf069_supplementary_data.zip › supplementary_materials_2-1_month-follow-up_of_2_cases.docx]
